# Linoleic Hydroperoxides Are Potent Hyperoxidative Agents of Sensitive and Robust Typical 2-Cys Peroxiredoxins

**DOI:** 10.3390/antiox14121422

**Published:** 2025-11-27

**Authors:** Vitória Isabela Montanhero Cabrera, Sabrina Vargas, Nathália Miranda de Medeiros, Gabrielle Nascimento Sividanes, Laura Fernandes da Silva, Larissa Regina Diniz, Thiago Geronimo Pires Alegria, João Henrique Ghilardi Lago, Marcos Hikari Toyama, Sayuri Miyamoto, Daniela Ramos Truzzi, Luis Eduardo Soares Netto, Marcos Antonio de Oliveira

**Affiliations:** 1 Instituto de Biociências, Universidade Estadual Paulista, UNESP, São Vicente 11330-900, SP, Brazil; vitoria.isabela@unesp.br (V.I.M.C.); sabrina.vargas@unesp.br (S.V.); laura.f.silva@unesp.br (L.F.d.S.); marcos.toyama@unesp.br (M.H.T.); 2 Departamento de Bioquímica, Instituto de Química, Universidade de São Paulo, São Paulo 05508-000, SP, Brazil; nathaliamiranda@usp.br (N.M.d.M.); dtruzzi@iq.usp.br (D.R.T.); 3 Departamento de Genética e Biologia Evolutiva, Instituto de Biociências, Universidade de São Paulo, São Paulo 05508-090, SP, Brazil; 4 Centro de Ciências Naturais e Humanas, Universidade Federal do ABC, Santo André 09210-180, SP, Brazil

**Keywords:** peroxiredoxin, hyperoxidation, long chain fatty acids hydroperoxides, enzyme inhibition

## Abstract

Typical 2-Cys peroxiredoxins (2-Cys Prxs, AhpC/Prx1 subfamily) are ubiquitous thiol peroxidases that efficiently reduce H_2_O_2_ and other hydroperoxides via a reactive peroxidatic Cys (C_P_). Under elevated hydroperoxide levels, C_P_ can be hyperoxidized to sulfinic (C_P_-SO_2_H) or sulfonic (C_P_-SO_3_H) acids, leading to enzyme inactivation. Notably, eukaryotic 2-Cys Prxs are orders of magnitude more sensitive to hyperoxidation (sensitive Prxs) by H_2_O_2_ than their bacterial counterparts (robust Prxs). Sensitivity to hyperoxidation also correlates with the catalytic triad composition: enzymes containing threonine (Thr-Prx) are more prone to hyperoxidation by H_2_O_2_ than those with serine (Ser-Prx). While hyperoxidation is reversed in eukaryotes by an enzyme (sulfiredoxin), it is generally considered irreversible in bacteria. Here, we compared the hyperoxidation susceptibility of three typical 2-Cys Prxs: human Prx2 (Thr-Prx, sensitive), *P. aeruginosa* (Thr-Prx, robust) and *S. epidermidis* (Ser-Prx, robust) to lipid hydroperoxides derived from linoleic acid, containing one or two peroxide moieties per molecule. Employing structural analysis, molecular simulations and kinetic assays, we found that lipid peroxides proved to be potent hyperoxidizing agents for all 2-Cys Prx tested, inactivating the enzymes up to 10,000 times faster than H_2_O_2_. These results may have implications for understanding bacterial oxidative stress responses and antimicrobial resistance.

## 1. Introduction

Typical 2-Cys peroxiredoxins (2-Cys Prxs, members of the AhpC/Prx1 subfamily), known as AhpC in bacteria, are abundant thiol peroxidases found in eukaryotic and prokaryotic cells. Like all peroxiredoxins, 2-Cys Prx uses a highly reactive cysteine residue, the so-called peroxidatic Cys (C_P_), to decompose their substrates [1,2,3,4]. C_P_ takes part of a catalytic triad, composed by a Thr, which in some cases is substituted by a Ser, and an Arg. These Thr/Ser and Arg residues facilitate the orientation and activation of the hydroperoxide molecule (R-OOH) through a hydrogen bond network, enabling optimal C_P_ reactivity through an S_N_2 mechanism [5,6,7]. The peroxidase activity of Prx initiates with C_P_-S^-^ attacking an oxygen atom of the hydroperoxide, causing heterolytic cleavage of the O-O bond with the concomitant oxidation of C_P_-S^-^ to C_P_-SOH (cysteine sulfenic acid). Typical 2-Cys Prx possess a second Cys residue, the so-called resolving Cys (C_R_) [8], which forms an intermolecular disulfide (between the C_P_ of one monomer and the C_R_ of the other) [9,10,11,12]. To initiate a new catalytic cycle, this disulfide bond must be reduced, a task carried out by the thioredoxin system, comprising the thioredoxin and thioredoxin reductase enzymes, or by the AhpF enzyme in several bacteria [1,4,9,13,14].

**Figure 1:**
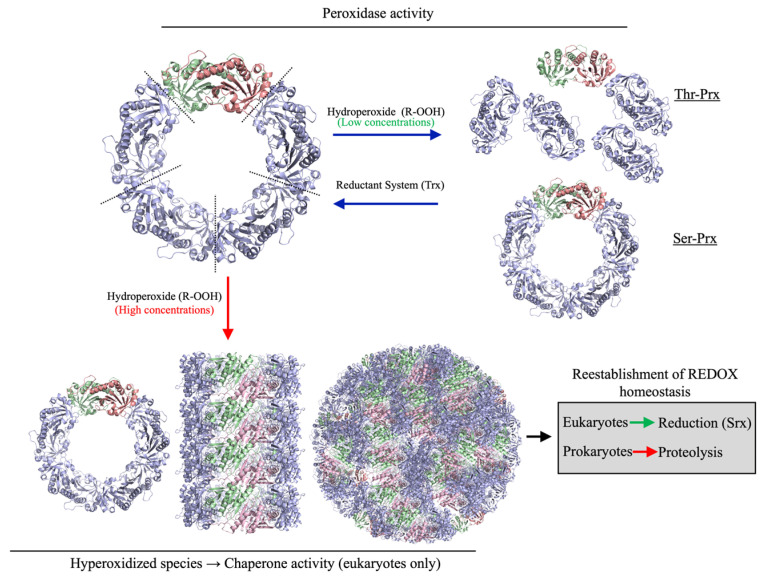
Functional and structural dynamics of 2-Cys Prxs. In their reduced state, 2-Cys Prx predominantly assemble as decamers. The disulfide formation in 2-Cys Prx favors decamer to dimer dissociation in Thr-Prx, but not in Ser-Prx. In their hyperoxidized state (C_P_-SO_2_H), 2-Cys Prxs lose their peroxidase activity and associate into very high molecular weight complexes. The reestablishment of redox homeostasis allows the reduction of C_P_-SO_2_H by sulfiredoxin in eukaryotes. Bacteria lack Srx; therefore, the hyperoxidized 2-Cys Prxs are proteolytically digested [20].

The basic oligomeric unit of typical 2-Cys Prxs is homodimeric, which under specific conditions, assemble into decameric (α_2_)_5_ ring-like structures [8]. The dynamic equilibrium between dimers and decamers is affected by several factors, such as protein concentration, redox state and pH [15,16]. In addition, the Thr/Ser polymorphism in the catalytic triad strongly influences the oligomeric state of typical 2-Cys Prx in the disulfide form. Enzymes with threonine (Thr-Prx) tend to dissociate into dimers, while those with serine (Ser-Prx) remain as decamers (Figure 1) [17,18,19]. Although some Ser-Prxs exist in eukaryotes, they are more prevalent in bacteria [17]. Despite sharing high structural similarity, eukaryotic Prxs and prokaryotic AhpC enzymes exhibit structural and functional differences that impact their activity. In eukaryotes, the typical 2-Cys Prx possesses a central insertion within the polypeptide chain of a GGLP motif, and a C-terminal α helix extension, containing a YF motif, which delays disulfide formation, making these enzymes more susceptible to C_P_ hyperoxidation to C_P_-SO_2_H (cysteine sulfinic acid) by H_2_O_2_. As these peroxidases lose peroxidase activity at low H_2_O_2_ levels, they are referred to as “sensitive” typical 2-Cys Prxs [12]. In contrast, prokaryotic isoforms typically lack these motifs and the C-terminal extension, making them significantly more resistant to hyperoxidation and oxidative inactivation by H_2_O_2_ and are thus considered “robust” [12].

The hyperoxidized state (C_P_-SO_2_H) cannot be reversed by conventional reductant systems (Trx or AhpF). Eukaryotes contain sulfiredoxin (Srx) that reduces C_P_-SO_2_H back to C_P_-SOH in an ATP-dependent process [21,22,23]. The presence of this system further distinguishes eukaryotic from prokaryotic 2-Cys Prxs, as bacteria lack a Srx equivalent. Therefore, hyperoxidation of prokaryotic AhpCs results in irreversible inactivation. Strikingly, hyperoxidation of C_P_ triggers the formation of complexes with very high molecular weight (Figure 1), of which their functional meaning is still debatable. For eukaryotic 2-Cys Prxs, chaperone (holdase) activity is frequently associated with these high molecular weight species [22,24,25,26].

Besides H_2_O_2_, Prxs reduce peroxynitrite and alkyl hydroperoxides with high efficiency [27]. Among alkyl hydroperoxides, lipid hydroperoxides deserve to be highlighted because of their high cellular abundance. Mono- and especially polyunsaturated fatty acids (MUFAs and PUFAs) are susceptible to peroxidation, generating products that can act as important signaling molecules [28,29,30,31]. PUFAs are particularly prone to oxidation due to the weakened C-H bonds at their bis-allylic positions, which favors hydrogen atom abstraction and subsequent oxidation [32]. Oxidized lipids are commonly found as components of membrane phospholipids, and their release can be mediated by highly conserved phospholipases [33]. With the exception of human Glutathione peroxidase 4 (Gpx4) and Peroxiredoxin 6 (Prdx6) [34,35], all other thiol peroxidases require the release of the fatty acids from the membrane to reduce the corresponding fatty acid peroxide. These hydroperoxides, even at relatively low concentrations, are highly toxic to bacterial cells, damaging biological membranes, and potentially causing their rupture and cell death [36,37,38,39].

Arachidonic acid, a PUFA commonly found in eukaryotes, participates in inflammatory and anti-inflammatory signaling cascades, as a substrate for the enzymatic generation of hydroperoxides that are toxic to bacteria such as *Staphylococcus aureus* and *Pseudomonas aeruginosa* [36,40]. The mechanism underlying this toxicity involves lipid peroxidation. To defend themselves against this oxidative insult, bacteria display highly efficient thiol peroxidases (such as AhpE and Ohr) to reduce MUFA- and PUFA-derived peroxides with exceptional rate constants (10^7^–10^8^ M^−1^s^−1^) [40,41].

Although the structure and biochemistry of AhpCs are very well-characterized [20], their ability to reduce lipid hydroperoxides, derivatives from MUFA and PUFA, have not been investigated in detail. In contrast, the rate constants for the reduction of PUFA-derived hydroperoxides by human Prx3 (HsPrx3), a mitochondrial and mammalian orthologue of AhpC, was already determined. HsPrx3, is rapidly oxidized (10^7^ M^−1^s^−1^) and hyperoxidized (10^5^–10^7^ M^−1^s^−1^) by 15-HpETE and prostaglandin G2 (PGG2) [42].

However, to date, no work comparatively evaluated the efficiency of PUFA hydroperoxides to oxidize/hyperoxidize sensitive and robust 2-Cys Prx. Furthermore, no comparison on the reactions of PUFA hydroperoxides with Thr-Prx with Ser-Prx were carried out. Since 2-Cys Prxs act as virulence factors in some pathogenic bacteria [43,44,45,46,47,48], the hyperoxidation of bacterial AhpCs is a potential way to combat pathogens, weakening their defenses against the oxidative insults imposed by the host. In this context, we initially hypothesized here that PUFAs carrying multiple -OOH groups in a single molecule would be more effective in hyperoxidizing and inactivating 2-Cys Prx. Of note, PUFAs are very abundant in host cell membranes, being good substrates to lipoxygenases and cyclooxygenases that generate hydroperoxides [28,49].

In this study, we investigated the effects of hydroperoxides derived from linoleic acid containing one (Li-OOH_(1)_) or two (Li-OOH_(2)_) hydroperoxide groups per molecule on distinct 2-Cys Prxs. These enzymes were selected based on their resilience to hyperoxidation and the presence of either Ser or Thr in their catalytic triads. Specifically, we examined human Prx2 (HsPrx2; sensitive; Thr-Prx), AhpC from *P. aeruginosa* AhpC (PaAhpC; robust; Thr-Prx) and *S. epidermidis* (SeAhpC; robust; Ser-Prx) using biochemical approaches and molecular docking simulations. All three enzymes efficiently reduced both Li-OOH_(1)_ and Li-OOH_(2)_ substrates, exhibiting very low K_m_ values. In addition, these hydroperoxides rapidly inactivated the 2-Cys Prxs at low concentrations. Kinetics studies indicated that Li-OOH_(1)_ is a superior substrate for HsPrx2 in comparison to Li-OOH_(2)_. Notably, our findings suggest that linoleic acid-derived hydroperoxides hyperoxidized both eukaryotic and prokaryotic 2-Cys Prxs at rate constants that are 100–10,000 times higher than those observed for H_2_O_2_. Computational simulations revealed that Li-OOH_(1)_ and Li-OOH_(2)_ interacted with active site residues in all three enzymes with Gibbs free energies ranging from −5.0 to −6.6 kcal/mol, positioning the peroxide function close to C_P_ (~3.0–4.3 Å). Taken together, our data demonstrate that lipid hydroperoxides are biological substrates for typical 2-Cys Prxs and act as potent hyperoxidizing agents, leading to a strong inhibitory effect.

## 2. Materials and Methods

### 2.1. Materials

All the chemical compounds were purchased from Sigma-Aldrich (St. Louis, MO, USA). Hydroperoxides derived from linoleic acid were synthesized by photooxidation of linoleic acid in an O_2_-saturated atmosphere as previously described [50,51]. Briefly, 100 mg of linoleic acid was dissolved in 5 mL of chloroform containing 0.07 mM methylene blue and exposed to irradiation from a 500 W tungsten lamp for 3.5 h. The reaction was carried out in an ice bath under a continuous O_2_ flow. After irradiation, methylene blue was removed, and Li-OOH_(1)_ and Li-OOH_(2)_ were isolated using silica gel column chromatography. Specifically, the reaction products were loaded onto the column and eluted using a stepwise gradient of chloroform and methanol, varying the ratio from 97:3 to 90:10 (% *v*/*v*). The concentration of Li-OOH_(1)_ and Li-OOH_(2)_ were determined spectrophotometrically (λ = 234 nm, ε_234_ = 25,000 M^−1^ cm^−1^) [51] and confirmed by iodometry [52].

The plasmids to express the proteins of the Trx system from *Escherichia coli* (EcTrx/EcTrxA and EcTrxR/EcTrxB); the Trx system from *Saccharomyces cerevisiae* (ScTrx1 and ScTrxR1); Prx2 from *Homo sapiens* (HsPrx2); AhpC from *P. aeruginosa* (PaAhpC) and *S. epidermidis* (SeAhpC) were obtained as described previously (Table 1) [17,53,54]. The *E. coli* BL21 (*DE3*) strain (Lucigen, Middleton, WI, USA) was used in expression procedures.

### 2.2. Microbiological Culture Media

The culture media used for bacterial protein expression was LB (1% triptone; 0.5% yeast extract; 0.5% NaCl). Solid media were obtained by adding 2% bacteriological agar.

### 2.3. Expression, Purification and Quantification of Recombinant Proteins

*E. coli* BL21 (*DE3*) cells (Lucigen, Middleton, WI, USA) containing the vector cloned with target genes were inoculated separately into 20 mL of LB medium containing the appropriate antibiotic (ampicillin or kanamycin, 100 μg/mL) and grown for 16 h/37 °C/250 rpm in an orbital shaker. Subsequently, the culture was transferred to 1 L of fresh LB/Amp and grown to OD_600_ ~ 0.6. Then, IPTG was added to a final concentration of 0.3 mM. The expression was performed for 3 h/37 °C/250 rpm, and then the cells were harvested by centrifugation (20 min/4 °C/4.000 g) and resuspended in 50 mM Tris buffer (pH 7.4) containing NaCl (500 mM). Cell disruptions were performed by sonication (30% amplitude) and nucleic acids were removed using streptomycin sulfate ([Final] = 1%). The cell extracts were centrifuged for 40 min/4 °C/12.000 g, and the protein extracts were collected. Once the proteins were expressed containing a His-tag, purification was performed by immobilized metal affinity chromatography using His-Trap crude columns (Cytiva, Uppsala, Sweden) by imidazole gradient. The purification quality was assessed by SDS-PAGE (12%) under reducing conditions. After these procedures, the proteins were desalted by gel filtration chromatography using PD10 columns (Cytiva, Uppsala, Sweden) and concentrated by centrifugation (4.000 g/4 °C) using Ultracel YM-30 concentrator (Millipore, Bedford, MA, USA) to ~1–5 mg/mL. The enzymes concentrations were determined by absorbance at 280 nm, considering the molar extinction coefficients for each protein (Table 2) obtained by the ProtParam tool (https://web.expasy.org/protparam/) (accessed on 8 October 2025).

### 2.4. Evaluation of Peroxidase Activity of Typical 2-Cys Prx by NADPH Oxidation Coupled Assays

To evaluate the reduction of different substrates (H_2_O_2_, cumene hydroperoxide -CHP), Li-OOH_(1)_ and Li-OOH_(2)_), we employed the NADPH oxidation coupled assay using the *E. coli* Trx system (EcTrx and EcTrxR) for the analyses of the PaAhpC and SeAhpC peroxidase activities, as previously described [17,55] and *S. cerevisiae* Trx system (ScTrx1 and ScTrxR1) for investigating HsPrx2 peroxidase activity [56].

### 2.5. Determination of 2-Cys Prx Free Thiol Groups

Protein sulfhydryl groups were determined using 5,5′-dithio-bis (2-nitrobenzoic acid) (Ellman’s reagent, DTNB) as follows: 20 μM of AhpCs or HsPrx2 (in a 100 µL final volume) were mixed with 2 μL of DTNB (10 mM) in 30 mM Tris-HCl (pH = 7.4), 1 mM EDTA and 8 M urea buffer. The release of 2-nitro-5-thiobenzoic acid (TNB) was monitored at 412 nm and the amount of TNB released was calculated using the molar absorption coefficient (13,600 M^−1^ cm^−1^) [57] in order to obtain the percentage of reduced protein (>90%), to perform fluorescence kinetic approaches.

### 2.6. Determination of Oxidation or Hyperoxidation Rates by the Intrinsic Fluorescence of the 2-Cys Prx

Prior to experiments, enzymes were reduced using 5 mM DTT at 37° for 1h. Excess of DTT was removed using a PD-10 desalting column (Cytiva, Uppsala, Sweden) and argon gas was introduced into the headspace of the solution to remove the molecular oxygen. The DTNB assay (see above) confirmed effective enzyme reduction. Then, 0.5 μM of reduced PaAhpC, SeAhpC or HsPrx2 (buffer: 50 mM Tris, pH 7.4 containing 50 mM NaCl) was mixed with increasing concentrations of Li-OOH_(1)_ or Li-OOH_(2)_ in an Applied Photophysics model SX20 stopped-flow spectrophotometer (Applied Photophysics, Leatherhead, UK). Redox dependent intrinsic fluorescence changes were monitored (λ_ex_ = 280 nm; λ_em_ ≥ 330 nm) at 10 °C. Observed rate constants (*k_obs_*) were determined by fitting the stopped-flow data to single exponential functions. Apparent second-order rate constants were determined from the slope of *k_obs_* values plotted against hydroperoxide concentrations. The OriginLab 10.1.0.178 Software (https://www.originlab.com) was used to perform the calculations of the constants.

### 2.7. Evaluation of HsPrx2 Hyperoxidation by Western Blotting

Samples of HsPrx2 (3 μM) reduced by 5 mM DTT for 1 h/RT were desalted and treated with increasing molar equivalents concentrations of H_2_O_2_, Li-OOH_(1)_ or Li-OOH_(2)_ (5, 12.5, 25 and 100 µM) for 30 min at room temperature and then were applied in 12% SDS-PAGE under reducing conditions (+β-ME) and transferred to a nitrocellulose membrane. The negative control for hyperoxidation was a DTT-reduced sample, which was applied alongside the molecular mass marker (S2600 TrueColor High Range Protein Marker—Sinapse Biotechnology, São Paulo, Brazil). The membrane was stained by Ponceau and kept overnight in a blocking solution (5% milk/TBS with 0.1% Tween). Then, the membranes were incubated with the human anti-PRDX-SO_2/3_ polyclonal primary antibody (1:2000 dilution) (ab16951 Abcam; Cambridge, UK) for 2 h at room temperature. After washing, the membranes were incubated for 1 h with the secondary HRP-conjugated anti-rabbit (1:10,000 dilution) (Santa Cruz Biotechnology, Santa Cruz, CA, USA) and washed again and data were acquired using the Image Lab 5.1 software from ChemiDoc™ MP Imaging System (Bio-Rad, Hercules, CA, USA).

### 2.8. Statistical Analysis

All analyses were performed at least three times in triplicate. Results were represented as mean ± standard deviation (SD) using GraphPad Prism version 6.05 software (GraphPad Prism Software, San Diego, CA, USA).

### 2.9. Structural Modeling of PaAhpC and SeAhpC

Structural ab initio predicted three-dimensional structures models of PaAhpC and SeAhpC were generated using the Alphafold 2-Colab [58,59] with the sequences obtained from the UniProt database (PaAhpC: Q02UU0 and SeAhpC: Q5HRY1). Model reliability was assessed by the local distance difference test (LDDT), predicted template modeling (pTM) and interface-predicted template modeling (ipTM) scores, and models with the highest score were selected for further analysis using UCSF Chimera X software (Version 1.7.1, University of California San Francisco, San Francisco, CA, USA).

### 2.10. Peroxides Molecular Docking in Typical 2-Cys Prx Active Site

Docking simulations were performed using the theoretical coordinates of PaAhpC and SeAhpC and the crystallographic coordinates of HsPrx2 (7KIZ). Three-dimensional structures of long-chain lipid hydroperoxides were generated using Molview (https://molview.org/) (accessed on 8 October 2025). AutoDock Vina v1.2.x [60] was used for all the molecular docking simulations, targeting the microenvironment of decameric Prx structures. Grid boxes (20 × 20 × 20 Å) were centered on the active sites’ microenvironment, and 30 configurations were generated for each active site on the dimer interface.

Docking accuracy was validated by re-docking the ligand using identical parameters. UCSF Chimera [61] was used to analyze each ligand orientation, assessing viability based on: (1) the distance of the oxygen atom of the hydroperoxide and gamma sulfur atom of C_P_, (2) ligand-binding energies (ΔG in kcal/mol), and (3) position of the peroxide moiety relative to the H_2_O_2_ position in the active site pocket of *Aeropyrum pernix K1* ApTPx (obtained by soaking of protein crystals with H_2_O_2_) [62]. LigPlot^+^ was used to further analyze protein–ligand interactions for positively selected results [63].

## 3. Results

### 3.1. HsPrx2 and AhpCs Reduce Lipid Hydroperoxides

**Figure 2:**
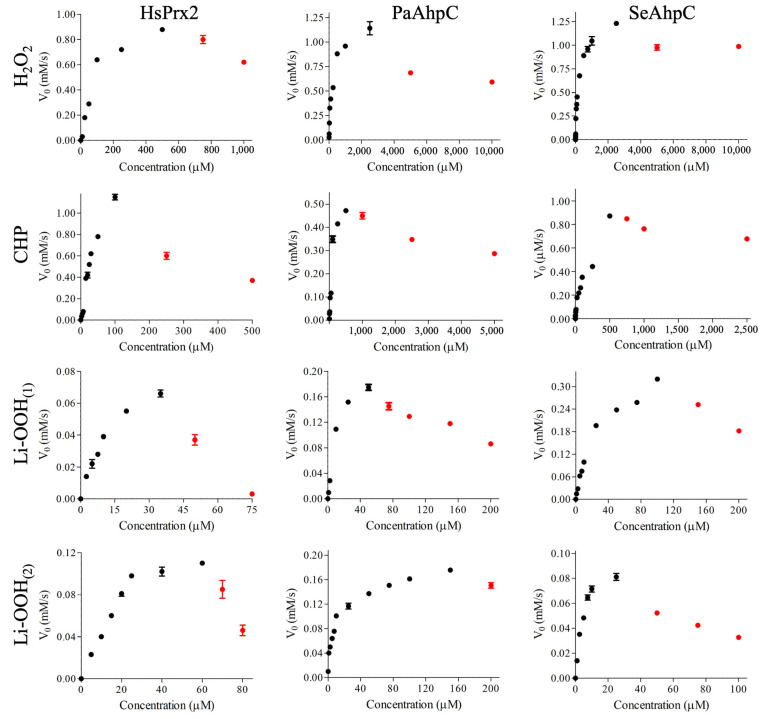
Steady-state analysis for the Trx linked peroxidase activity of HsPrx2, PaAhpC and SeAhpC over different kinds of peroxides (H_2_O_2_, CHP, Li-OOH_(1)_ or Li-OOH_(2)_). NADPH oxidation was monitored at 37 °C by the absorbance decrease (λ = 340 nm). Reactions mixtures containing AhpC (3.0 µM) were performed using EcTrx (6.0 µM), EcTrxR (0.9 µM), NADPH (150 µM), HEPES (50 mM, pH = 7.4), 100 µM DTPA and 1 mM sodium azide. Reactions containing HsPrx2 were performed using the yeast Trx system under the following conditions: HsPrx2 (3.0 µM), ScTrx1 (6.0 µM), ScTrxR1 (0.9 µM), NADPH (150 µM), HEPES (50 mM, pH 7.4), 100 µM DTPA, and 1 mM sodium azide. The assays were started by the addition of increasing peroxide concentrations. The enzymatic parameters were obtained by non-linear regression of the phase corresponding to low hydroperoxide concentrations (black dots). The red plots correspond to the inhibition resulting from hyperoxidation. The experiments were performed three times in triplicate with similar results.

To assess the peroxidase activities of typical 2-Cys Prxs towards lipid hydroperoxides, we conducted NADPH coupled assays using heterologous Trx systems from *S. cerevisiae* or *E. coli* (Figure 2). In addition to Li-OOH_(1)_ and Li-OOH_(2),_ we also determined the kinetic parameters for H_2_O_2_ and the CHP, a synthetic compound commonly used to evaluate the peroxidase activity of Prx over organic substrates.

Non-linear regression with the Michaelis–Menten equation, using data from the initial, linear phase at lower hydroperoxide concentrations, revealed that all three 2-Cys Prxs displayed significantly lower K_m_ values for lipid hydroperoxides (Li-OOH_(1)_ (~16.5–27 µM) and Li-OOH_(2)_ (~4.5–23 µM) than for H_2_O_2_ and CHP (~105–178 and ~57–82 µM, respectively), indicating that these peroxidases present higher affinity for lipid hydroperoxides (Figure S1 and Table 3). In contrast, *k*_cat_ values for H_2_O_2_ (~0.31–0.42 s^−1^) and CHP (~0.18–0.56 s^−1^) were considerably higher than those for lipid hydroperoxides (~0.03–0.32 s^−1^). Consequently, *k*_cat/_ K_m_ values were similar for the distinct peroxides (Figure S1, Table 3).

Concerning the inactivation of the peroxidases by C_P_ hyperoxidation (C_P_-SO_2/3_) [12,17], the amount of H_2_O_2_ (~750 μM) and CHP (250 μM) required to decrease the rates of NADPH oxidation by HsPrx2 were considerably lower than the amount of peroxides required to inhibit bacterial PaAhpC (Thr-Prx) and SeAhpC (Ser-Prx) (~5000 μM/H_2_O_2_ and 1000 μM/CHP), as expected [64] (Figure 2).

In relation to lipid hydroperoxides, the amounts required to decrease the rates of NADPH oxidation were markedly lower for all typical 2-Cys Prxs. In the case of HsPrx2, Li-OOH_(2)_ at approximately 50 μM and Li-OOH_(1)_ at around 70 μM significantly inhibited the peroxidase activity_._ Remarkably, very low levels of Li-OOH_(1)_ and Li-OOH_(2)_ were sufficient to inactivate the robust bacterial 2-Cys Prxs that were resilient to hyperoxidation by H_2_O_2_ and CHP. For PaAhpC, inhibition of NADPH oxidation occurred at 75 μM (Li-OOH_(1)_) and 200 μM (Li-OOH_(2)_), whereas for SeAhpC, similar effects were observed at 150 μM (Li-OOH_(1)_) and approximately 50 μM (Li-OOH_(2)_). Therefore, inactivation occurred with comparable potency between Thr-Prx and Ser-Prx groups (Figure 2). Overall, minimal amounts of lipid hydroperoxides were sufficient to inactivate typical 2-Cys Prxs, regardless of sensitivity or robustness, belonging to Thr-Prx or to Ser-Prx groups. Nevertheless, the presence of two peroxide moieties in Li-OOH_(2)_ did not render this compound more effective in hyperoxidizing 2-Cys Prxs than Li-OOH_(1)_.

These findings are particularly important, as this represents the first comparative study employing lipid hydroperoxides, revealing that all typical 2-Cys Prxs analyzed here, with distinct features, are susceptible to hyperoxidation even at very low lipid hydroperoxide levels.

### 3.2. Assessing HsPrx2 C_P_ Hyperoxidation by Immunoblotting

**Figure 3:**
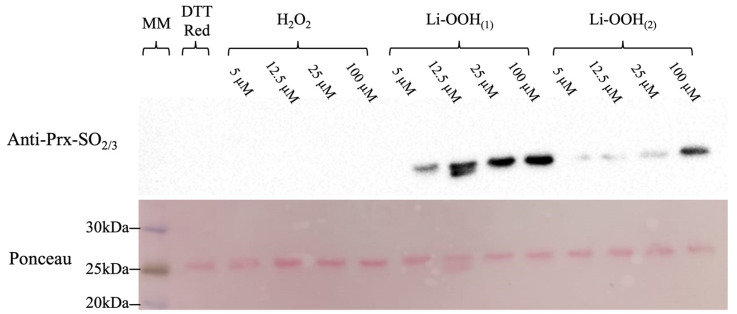
Assessing C_P_ hyperoxidation by immunoblotting. Pre-reduced samples of HsPrx2 were treated with increasing concentrations of H_2_O_2_, Li-OOH_(1)_ or Li-OOH_(2)_ (1, 2.5, 5 or 20 molar equivalents; 5, 12.5, 25 or 100 µM) (1 h/37 °C) and resolved in 12% SDS-PAGE under reducing conditions (β-mercaptoethanol 200 mM) to avoid the presence of dimers containing one hyperoxidized C_P_ and one intermolecular disulfide. A DTT-reduced sample was used as a hyperoxidation negative control. The samples were transferred to a membrane (Cytiva) using the Trans-Blot turbo (Biorad) at 30 °C/20 min. The membrane was kept overnight in a blocking solution (5%) and then incubated in a TBS-Tween solution containing anti Prx-SO_2/3_ (1:2000 dilution) for 2 h. After washing, the membrane was incubated for 1 h with HRP-conjugated anti-rabbit (1:10,000 dilution), washed again, and data were acquired using ChemiDoc System/Image (Biorad) (upper panel). Results are from one of three independent experiments with similar findings.

To evaluate the C_P_ hyperoxidation, we performed immunoblotting using the human anti-SO_2_/_3_, exposing the samples to increasing concentrations of H_2_O_2_ (control) or lipid hydroperoxides. After oxidation, the samples were resolved in SDS PAGE under reducing conditions (e.g., β-mercaptoethanol). This procedure prevents the detection of dimers containing hyperoxidized C_P_ and one intermolecular disulfide, thereby facilitating the detection of the hyperoxidized species in one single band. In the conditions tested, only Li-OOH were able to hyperoxidize HsPrx2 (Figure 3). Accordingly with the NADPH coupled assay, Li-OOH_(1)_ hyperoxidized HsPrx2 at a higher extent than the Li-OOH_(2),_ while H_2_O_2_ did not hyperoxidize this 2-Cys Prx. We also tested bacterial isoforms. Nevertheless, the heterologous nature of the antibody did not yield reliable results.

### 3.3. Determination of Hyperoxidation Rates by Intrinsic Trp Fluorescence

Since Li-OOH_(1)_ and Li-OOH_(2)_ rapidly hyperoxidized and inactivated 2-Cys Prxs, we aimed to determine the hyperoxidation rates of the enzymes by following redox dependent changes in the intrinsic Trp fluorescence. In this method, the very rapid oxidation and hyperoxidation of 2-Cys Prxs is followed in a stopped-flow equipment attached to a fluorescence detector. The fluorimetric profile is composed of a first phase, in which a fast drop in fluorescence intensity is observed, which has been ascribed to the oxidation of C_P_ in 2-Cys Prx, followed by a second phase of raising in fluorescence intensity attributed to the hyperoxidation [41,42,65].

**Figure 4:**
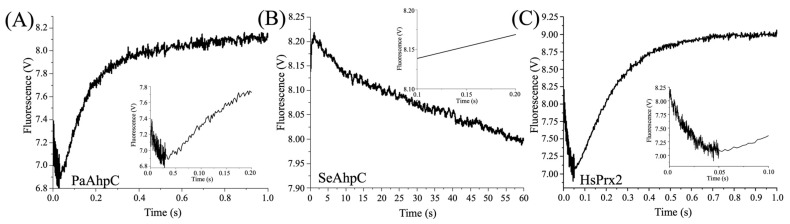
Fluorescence profiles of bacterial and human 2-Cys Prx (0.5 μM) oxidized with 5 uM Li-OOH_(1)_.The graphics show the fluorescence profiles of (**A**) PaAhpC, (**B**) SeAhpC and (**C**) HsPrx2. The inserts in the figures highlight the first 0.1–0.2 s of the reactions. The intrinsic fluorescence changes in the protein were monitored (λ_ex_ = 280 nm; λ_em_ = 330 nm) at 10 °C in a spectrofluorometer coupled to stopped flow and performed in triplicate at least three times.

Unfortunately, it was not possible to determine the oxidation rates for bacterial AhpCs. In the case of PaAhpC, the fluorescence decays of the first phase were extremely fast in the first 0.025 s (Figure 4A and insert). To SeAhpC, the fluorescence profile was not compatible with this technique, since it was very slow (>60 s) (Figure 4B). For HsPrx2, the fluorescence profile displayed both phases, enabling analysis of the corresponding kinetic parameters (Figure 4C and insert).

**Figure 5:**
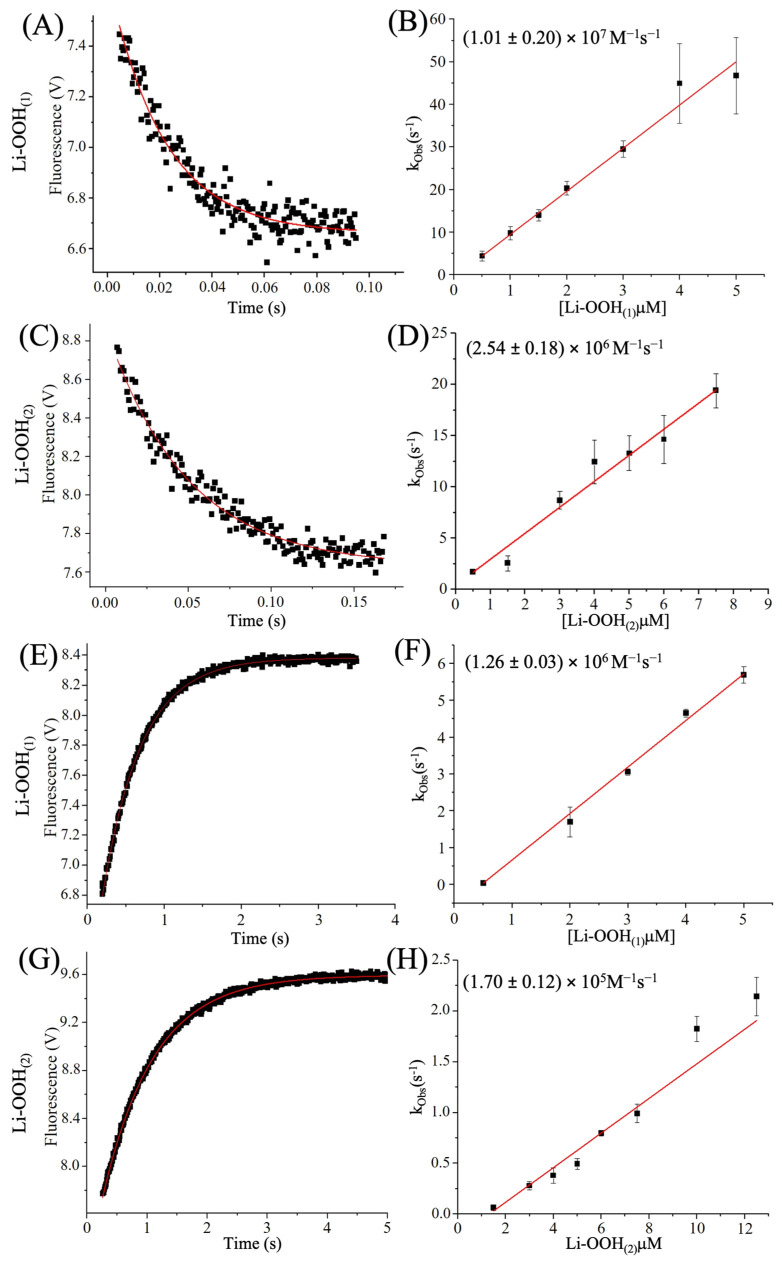
Determination of second order rate constants of HsPrx2 oxidation and hyperoxidation by Li-OOH_(1)_ and Li-OOH_(2)_. The protein samples were prepared as described in Material and Methods section. The graphics (**A**,**C**,**E**,**G**) show the fluorescence profiles of HsPrx2 (fixed concentration of 0.5 μM) oxidized with 5 μM of Li-OOH_(1)_ and Li-OOH_(2)_. In figure (**A**), the oxidation profile of the enzyme by Li-OOH_(1)_ and (**C**) Li-OOH_(2)_ is shown, while (**E**,**G**) show the hyperoxidation profile of HsPrx2. All experiments were repeated 3 times and carried out in triplicate. The apparent second-order rate constants were determined from the slope of *k_obs_* values plotted against hydroperoxide concentrations. In (**B**), the *k*_-LiOOH(1)_oxidation_ and (**D**) the *k-*_Li-OOH(2)_oxidation_ graphs are represented, and in (**F**), the *k*_-LiOOH(1)_hyperoxidation_ and (**H**) *k*_-Li-OOH(2)_hyperoxidation_ graph. The OriginLab 10.1.0.178 Software was used to perform the calculations of the constants.

Despite the very rapid fluorescence decay observed for HsPrx2, with Li-OOH_(1)_ and Li-OOH_(2)_ (Figure 5A,C), we are able to determine the second-order oxidation constants to be (1.01 ± 0.20) × 10^7^ M^−1^ s^−1^ and (2.54 ± 0.18) × 10^6^ M^−1^ s^−1^, respectively (Figure 5B,D). The hyperoxidation second-order rate constants for HsPrx2 were determined as 1.26 ± 0.03 × 10^6^ M^−1^s^−1^ for Li-OOH_(1)_ (Figure 5E,F) and 1.70 ± 0.12 × 10^5^ M^−1^s^−1^ for Li-OOH_(2)_ (Figure 5G,H). These findings align with steady-state kinetics (Figure 2 and Table 3) and immunoblotting data (Figure 3), which collectively indicate greater hyperoxidation efficiency with Li-OOH_(1)_ compared to Li-OOH_(2)_. Notably, these rates are 10- to 100-fold higher than those reported for H_2_O_2_-induced hyperoxidation of HsPrx2 (~ 10^4^ M^−1^s^−1^) [66].

**Figure 6:**
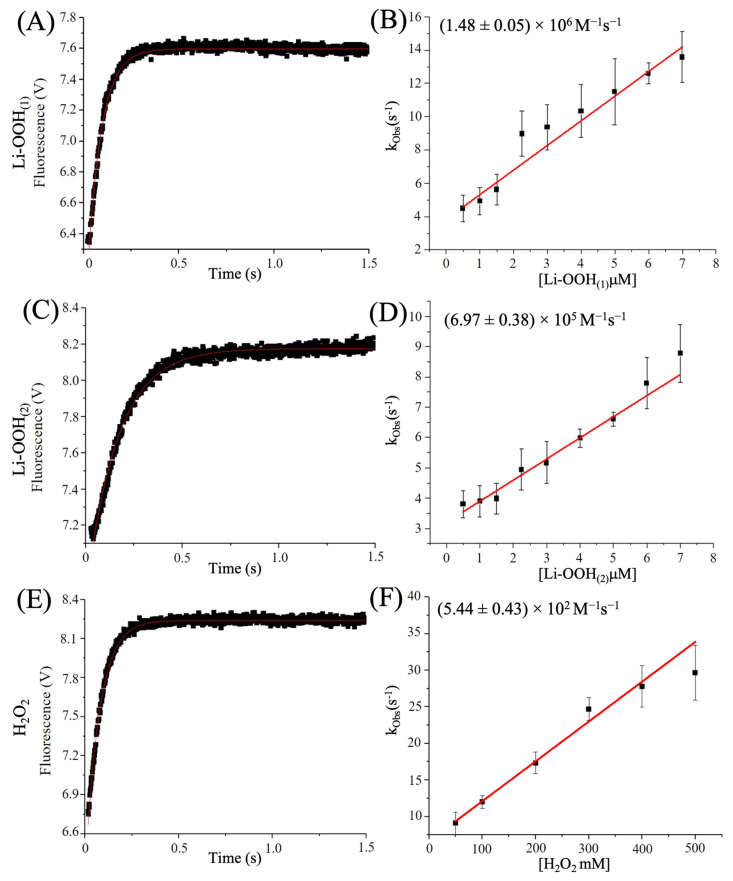
Second-order rate constants determination of PaAhpC hyperoxidation by Li-OOH_(1)_, Li-OOH_(2)_ and H_2_O_2_. The previously reduced enzyme was mixed with increasing concentrations of LiOOH_(1)_, Li-OOH_(2)_ or H_2_O_2_, in a stopped-flow spectrophotometer (Applied Photophysics SX20) and the intrinsic fluorescence changes were monitored (λ_ex_ = 280 nm; λ_em_ ≤ 330 nm) at 10 °C. The graphics show the fluorescence profiles of PaAhpC (fixed concentration of 0.5 μM) oxidized with Li-OOH_(1)_ (5 μM) (**A**), Li-OOH_(2)_ (5 μM) (**C**) or H_2_O_2_ (100 mM) (**E**). The experiments were carried out in triplicate at 10 °C and repeated at least three times. The apparent second-order rate constants were determined from the slope of k*_obs_* values plotted against increasing hydroperoxide concentrations: (**B**) k-_LiOOH(1)_hyperoxidation,_ (**D**) k-_Li-OOH(2)_hyperoxidation_ and k-_H2O2_hyperoxidation_ (**F**) graphs. OriginLab 10.1.0.178 Software was used for data analysis.

For PaAhpC, second-order hyperoxidation rate constants were 1.48 ± 0.05 × 10^6^ M^−1^s^−1^ with Li-OOH_(1)_ (Figure 6A,B) and 6.97 ± 0.38 × 10^5^ M^−1^s^−1^ with Li-OOH_(2)_ (Figure 6C,D). In contrast, hyperoxidation by H_2_O_2_ (Figure 6E,F) are three-to-four orders of magnitude lower than lipid hydroperoxides, yielding markedly lower rate constants (5.44 ± 0.43 × 10^2^ M^−1^s^−1^) (Figure 6E,F).

Together, our data shows that lipid hydroperoxides inactivate 2-Cys Prx sensitive or robust with similar rates (~10^5–6^ M^−1^s^−1^). Data concerning the rate constants of oxidation and hyperoxidation of this work and others are summarized in Table 4.

### 3.4. Ligand–Enzyme Interactions Simulations by Computer-Assisted Analysis

**Figure 7:**
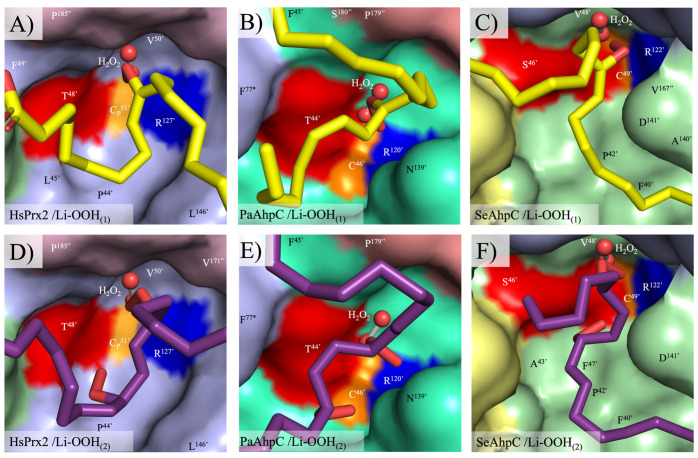
Best docking conformation of Li-OOH_(1)_ and Li-OOH_(2)_ at the active sites of HsPrx2 (crystal structure) and PaAhpC/SeAhpC (theoretical models). The active sites are located at the dimer–dimer interface of the decamer. The best configurations Li-OOH_(1)_ (carbons in yellow) and Li-OOH_(2)_ (carbons in purple) docked in the active site pockets of the HsPrx2, PaAhpC and SeAhpC are depicted in (**A**–**C**) and (**D**–**F**), respectively. Residues of the intimate dimer containing the catalytic triad are marked with a prime (’) for one monomer and with a quotation mark (”) for the complementary monomer. Amino acids from the adjacent homodimer are assigned with an asterisk (*). The catalytic triad residues are colored in red (Thr/Ser), yellow (C_P_) and blue (Arg). The molecular graphics were generated using Pymol 2.4.0.

To understand the structural basis for the extremely fast oxidation and hyperoxidation of typical 2-Cys Prxs by lipid peroxides, molecular docking analyses were performed. The crystallographic structure of HsPrx2 (1KIZ) and theoretical decameric models of the bacterial isoforms in reduced state were used. The docking results were evaluated by comparing the positioning of the ligands with that of H_2_O_2_ in the active site of ApTPx from *Aeropyrum pernix K1* [62]. The predicted binding conformations of Li-OOH_(1)_ and Li-OOH_(2)_ were closely aligned with the H_2_O_2_ molecule present in *A. pernix* crystal structure and were near the catalytic triad (Figure 7).

**Figure 8:**
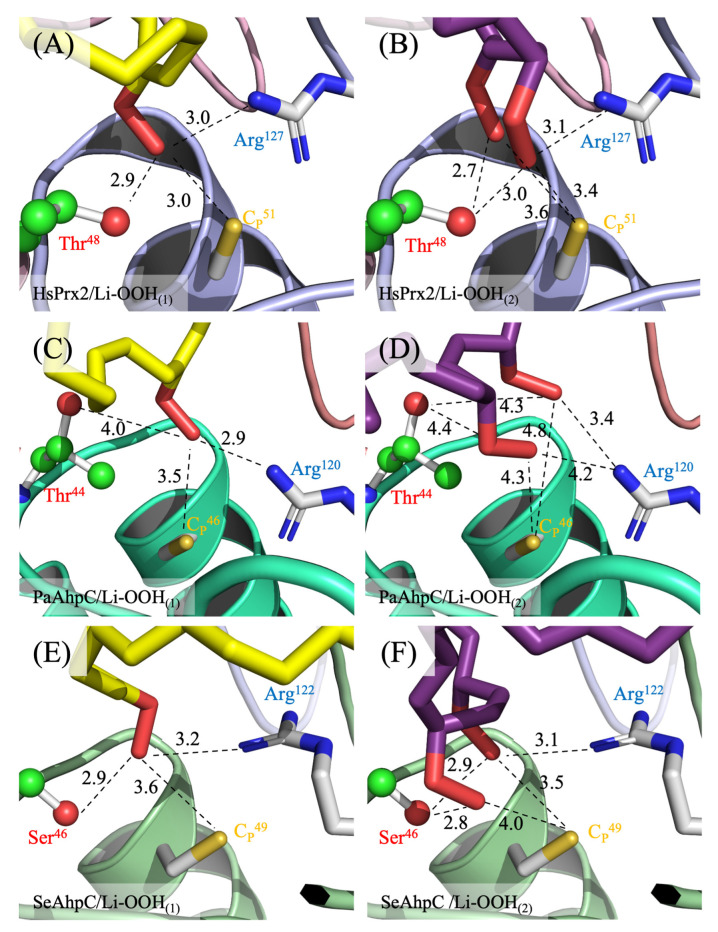
Molecular interactions among the residues of the active sites and the best conformation of lipid hydroperoxides. (**A**) HsPrx2/Li-OOH_(1)_, (**B**) HsPrx2/Li-OOH_(2)_, (**C**) PaAhpC/Li-OOH_(1)_, (**D**) PaAhpC/Li-OOH_(2)_, (**E**) SeAhpC/Li-OOH_(1)_ and (**F**) SeAhpC/Li-OOH_(2)_. Distances in Angstroms (Å) are represented by dashed black lines. The HsPrx2 and AhpC structures are in cartoon and the catalytic triad residues, C_P_ and Arg, and the peroxides are represented by sticks with carbons (C) colored in white. The Thr-Ser polymorphism is depicted by balls and sticks with C in green and the caption in red. The C atoms of the Li-OOH_(1)_ and Li-OOH_(2)_ are colored in yellow and purple, respectively. Oxygen (O) and nitrogen (N) are in red and blue. The figures were generated using PyMol 2.4.0.

The distances between the reactive groups of the catalytic triad residues and the peroxide ligands vary slightly among residues and enzymes but remain consistently close to the peroxide functional group (Thr^Oγ^/Ser^Oγ^ ~ 2.7–4.4; C_P_^Sγ^ ~ 3.0–4.8 and Arg^Nε^ ~ 2.9–4.2 Å), which would, in principle, allow peroxide reduction (Figure 8). The peroxide molecules exhibited strong stabilization within the active site pockets of HsPrx2 and AhpCs enzymes (PaAhpC and SeAhpC) with Gibbs free energies (ΔG), ranging from −5.2 to −5.7, −5.0 to −5.7 and −6.4 to −6.6 kcal/mol, respectively. This stabilization is mediated by numerous apolar interactions and salt bridges with conserved residues within the enzymes, including with residues of the catalytic triad (Figure S3). None of the conformations observed for Li-OOH_(1)_ and Li-OOH_(2)_ with Thr-Prx (HsPrx2 and PaAhpC) were significantly more favorable than those with Ser-Prx (SeAhpC). This observation suggests that both substrates display comparable affinities and stabilities across these enzyme groups. The data summarizing the optimal ligand-binding conformation for HsPrx2, PaAhpC and SeAhpC are presented in Table 5 and are consistent with the kinetic data, indicating that Li-OOH_(1)_ and Li-OOH_(2)_ interact similarly with both types of 2-Cys Prx enzymes.

In summary, the structural data align with the kinetic findings, indicating that fatty acid hydroperoxides (Li-OOH_(1)_ or Li-OOH_(2)_) are very good substrates for all types of 2-Cys Prxs. They potently inactivated peroxidase activities, including in enzymes that are otherwise resistant to H_2_O_2_-induced hyperoxidation.

## 4. Discussion

The typical 2-Cys Prx from eukaryotes and prokaryotes were described almost simultaneously around forty years ago by independent research groups using different methodologies [67,68]. With the growing number of studies, it has become evident that they exhibit a ubiquitous distribution among organisms and extraordinary activity over H_2_O_2_, peroxynitrite and organic hydroperoxides [64,69,70,71]. However, peroxides at elevated concentrations inhibit the peroxidase activities of these enzymes by C_P_ hyperoxidation (C_P_-SO_2_H), as a consequence of C_P_ reaction with two hydroperoxide molecules before disulfide bond formation [12].

Investigations on the sensitivity to inactivation have revealed structural diversity among typical 2-Cys Prx enzymes. In eukaryotes, the sensitive enzymes contain insertions of hydrophobic motifs and C-terminal extensions that favor inactivation by hyperoxidation, while in prokaryotes, these elements are absent and the enzymes exhibit higher resistance to inactivation by H_2_O_2_, and are so-called robust [12]. More recently, it has been demonstrated that a natural polymorphism of the catalytic triad, the replacement of Thr by a Ser, leads to functional and structural alterations including differences in sensitivity to hyperoxidation by H_2_O_2_, with Thr-Prx being more sensitive than Ser-Prx [17,18,19].

2-Cys Prxs display enhanced sensitivity to hyperoxidation by organic hydroperoxides than by H_2_O_2_. However, the rate constants for reactions with biologically relevant organic hydroperoxides have only recently been determined for typical 2-Cys Prx—for instance, urate hydroperoxide with the bacterial isoform AhpC from *X. fastidiosa* [47], and to urate and arachidonic acid hydroperoxides with human isoforms [42,65]. To date, however, no comparative analysis has been carried out across the different classes of typical 2-Cys Prxs.

Although AhpC has been originally described as a factor involved in the decomposition of linoleic acid hyperoxide in partially purified samples [67], no work to date has systematically investigated its reactivity on this type of substrate. In addition, under oxidative stress, hydroperoxides can be generated from polyunsaturated lipids with more than one peroxide moiety [72,73]. This is an important aspect, since a single hydroperoxide molecule can, in principle, oxidize and hyperoxidize these enzymes.

The present study comparatively evaluated the affinity and susceptibility to hyperoxidation of three types of typical 2-Cys Prx: HsPrx2 (Thr/sensitive), PaAhpC (Thr/robust), and SeAhpC (Ser/robust). We used lipid hydroperoxides containing one or two peroxide groups, Li-OOH_(1)_ and Li-OOH_(2),_ as substrates. Enzymatic assays revealed that all enzymes decompose both peroxides, presenting an apparent K_m_ lower than those determined for H_2_O_2_. Notably, low amounts of lipid hydroperoxides were sufficient to inhibit the peroxidase activity through hyperoxidation (Figure 2). Moreover, Li-OOH_(1)_ and Li-OOH_(2)_ were equally effective in inactivating both HsPrx2 and AhpCs, suggesting that the presence of two peroxide groups in the substrate did not enhance hyperoxidation. As we compared the same typical 2-Cys Prx with distinct peroxides, the use of distinct reductive systems does not affect the validity of the comparisons.

Western blot analyses revealed that the hyperoxidation of HsPrx2 induced by lipid peroxides was greater than that induced by H_2_O_2_. Furthermore, Li-OOH_(1)_ was more effective hyperoxidizing agent than Li-OOH_(2)_. To further evaluate oxidation and hyperoxidation kinetics, rapid-mixing approaches were employed. For HsPrx2, the rate constants for oxidation were remarkably high (~10^7^ and 10^6^ M^−1^s^−1^ for Li-OOH_(1)_ and Li-OOH_(2)_, respectively). Similarly, the hyperoxidation rate constants for lipid peroxides were also elevated for Li-OOH_(1)_ and Li-OOH_(2)_ (~10^6^ and 10^5^ M^−1^s^−1^, respectively).

Regarding the bacterial proteins, it was not possible to determine the oxidation rate constants due to very fast reactions, suggesting rates higher than 10^7−8^ M^−1^s^−1^ (PaAhpC-ThrPrx), or due to fluorescence anomalies (SeAhpC). On the other hand, the hyperoxidation second order rate constants of Li-OOH_(1)_ and Li-OOH_(2)_ to PaAhpC were determined as ~10^6^ and 10^5^ M^−1^s^−1^, respectively, closely matching those obtained for HsPrx2 (Table 4).

It is important to note that when H_2_O_2_ was used as oxidizing substrate, the rates of hyperoxidation are of the order of 10^4^ M^−1^s^−1^ for the sensitive HsPrx2 and 10^2^ M^−1^s^−1^ for the robust PaAhpC. However, when the substrate is a lipid peroxide, both 2-Cys Prxs are sensitive to hyperoxidation, indicating that this classification and the mechanisms involved vary according to the oxidizing substrate.

Our data are in agreement with those obtained by Cardozo and colleagues for Prx3, where the rates of hyperoxidation by lipid peroxides derived from arachidonic acid (15-HpETE and PGG2) were determined as 10^7^ M^−1^s^−1^ [42], much higher than those observed to H_2_O_2_ (10^4^ M^−1^s^−1^) [66]. Interestingly, when CHP was used as a substrate for HsPrx2, the oxidation rate constants were very high (2.43 ± 0.05 × 10^8^ M^−1^s^−1^), but the hyperoxidation rate constants were quite low (5.91 ± 0.19 × 10^3^ M^−1^s^−1^) (Figure S2), indicating that structural features of the biological substrates are involved in the effectiveness of 2-CysPrx hyperoxidation.

Another relevant feature is that the hyperoxidation rate constants for Li-OOHs were substantially higher than those for H_2_O_2_. Specifically, the constants for HsPrx2 were 100 and 10 times higher with Li-OOH_(1)_ and Li-OOH_(2)_, respectively, and for PaAhpC, they were up to 10,000 and 1000 times higher (Table 4). It is also important to note that our experiments were conducted at 10 °C, whereas those reported in the literature were performed at approximately 20 °C. This suggests that the rate constants for the hyperoxidation of 2-Cys Prxs by Li-OOHs are likely even higher than the corresponding hyperoxidation rate constants reported for other peroxides (Table 4).

Notably, the rate constants for Li-OOH_(1)_ were higher than those for Li-OOH_(2)_, which is in line with other biochemical approaches used in this work. Aiming to shed a light on this question, we perform molecular docking simulations, but the results indicated that both peroxides can be productively stabilized in all the typical 2-Cys Prxs active sites with the hydroperoxide functions of Li-OOH_(1)_ and Li-OOH_(2)_ in close vicinity to C_P_ (Figure 7 and Figure 8). In principle, this could favor oxidation (Li- OOH_(1)_ and (Li-OOH_(2)_) or fast hyperoxidation (Li-OOH_(2)_) of the enzymes.

Therefore, the reason for differences in the oxidation/hyperoxidation rates is still unclear, but molecules containing more than one peroxidation have more than one reactive group and these can react with each other to form secondary radical and non-radical compounds as endoperoxides, which, in principle, explains the lower reactivity of the enzyme, either as consequence of enzyme damage or as a result smaller amount of substrate to decompose [74,75].

Furthermore, the high reactivity of Prxs to hydroperoxides is related to its capacity to stabilize transition states of nucleophilic substitution (S_N_2) reactions, where the S^γ^ of C_P_ and the two oxygen atoms of the hydroperoxides are aligned in a straight line [6]. Possibly, in the case of Li-OOH_(2)_ substrates, one peroxide function can interfere with the other, making it more difficult for the molecules to achieve the transition state. The docking simulations (Figure 8B,D,F) suggest that this is indeed the case, contributing to the lower reactivities of 2-Cys Prxs towards Li-OOH_(2)_ (Table 4). It is also important to note that both the docking simulations and steady-state kinetics showed no significant difference in Lp-OOH affinity between typical 2-Cys Thr-Prx and Ser-Prx, indicating it is a high-affinity substrate for both enzyme groups. However, future studies require a Ser-Prx compatible with the fluorescence methodology to reach an unequivocal conclusion.

The results described in this study and in the work by Cardozo and colleagues [42] show that fatty acid hyperoxides are powerful hyperoxidizing agents for typical 2-Cys Prx, and are even superior than the H_2_O_2_, which is considered a universal substrate for Prxs. The inhibition of the peroxidase activity of typical 2-Cys Prxs has an impact on the physiology of the cells. For H_2_O_2_-sensitive isoforms, present in eukaryotes, hyperoxidation is considered an evolutionary gain, making possible the signal transduction by hydroperoxides with implications in cell growth, transcriptional regulation, defense against oxidative damage and other processes [76,77,78,79]. Lipid hydroperoxides may be important in promoting a similar mechanism in bacteria with still-unknown implications in prokaryote cell signaling. The high hyperoxidation rates of lipids hydroperoxides open the possibility that these molecules could hyperoxidate/inactivate these enzymes under physiological conditions. In this context, it is tempting to hypothesize that lipid peroxides may act as biological inhibitors of the peroxidase activity of typical 2-Cys Prx. Additionally, since 2-Cys Prx are involved in genetic and infectious diseases, the knowledge of biological organic substrates may help in the identification of inhibitors that share functional and structural characteristics with biological oxidizing substrates. In fact, recently we identified one natural prenylated benzoic acid from *Piper crassinervium,* which can inhibit PaAhpC peroxidase activity [80]. Notably, among the functional groups of the compound, two of them resemble PUFA hydroperoxides: a hydrophobic tail and a carboxylic group.

## 5. Conclusions

Our results revealed that lipid hydroperoxides are not only substrates to different classes of 2-Cys Prx but also biological substrates capable of hyperoxidizing and inactivating the peroxidase function of both humans and bacteria enzymes at a similar extend. The knowledge of these organic biological substrates may provide a better understanding of biological roles of typical 2-Cys Prx and may support the selection of leading compounds that act as Prx inhibitors.

## Figures and Tables

**Table 1:** Expression plasmids used in this work.

Plasmid	Antibiotic Resistance *	Reference
pET15b::*ec_trx*	Amp	[17]
pET15b::*ec_trxr*	Amp	[17]
pET15b::*pa_ahpc*	Amp	[17]
pET15b::s*e_ahpc*	Amp	[17]
pET17b::*sc_trx1*	Amp	[53]
pPROEX::*sc_trxr1*	Kan	[53]
pET28a::*hs_prx2*	Kan	[54]

* Abbreviations: Amp, ampicillin; Kan, kanamycin.

**Table 2:** Molar extinction coefficients and molecular weight of enzymes used in this study.

Protein	Ɛ 280 nm (M^−1^ cm^−1^)	Molecular Weight (kDa)	Uniprot Entry
PaAhpC	22.460	22.82	Q02UU0
SeAhpC	26.930	23.36	Q5HRY1
EcTrx	15.470	14.09	P0AA25
EcTrxR	20.400	36.90	P0A9P4
HsPrx2	21.555	23.92	P321194
ScTrx1	9.970	11.23	P22217
ScTrxR1	30.370	37.33	P29509

**Table 3:** Kinetic parameters for HsPrx2 and bacterial AhpCs with various peroxides were determined. The calculations used only the initial rates (v_0_) from the ascending of the curves, which correspond to low hydroperoxide concentrations ^a^.

	Hpx	K_m_ (µM)	*k*_cat_ (s^−1^)	V_max_ (µM/s^−1^)	*k*_cat_/K_m_ (M^−1^ s^−1^)
**HsPrx2**	H_2_O_2_	105 (±18)	0.35 (±0.02)	1.07 (±0.05)	3.4 (±0.9) × 10^3^
	CHP	57 (±17)	0.56 (±0.09)	1.68 (±0.09)	9.8 (±1.3) × 10^3^
	Li-OOH_(1)_	16.5 (±1)	0.19 (±0.10)	0.10 (±0.01)	1.2 (±0.2) × 10^3^
	Li-OOH_(2)_	23 (±6)	0.32 (±0.01)	0.16 (±0.01)	1.4 (±0.4) × 10^3^
**PaAhpC (Thr)**	H_2_O_2_	116 (±13)	0.31 (±0.01)	0.67 (±0.02)	2.8 (±0.2) × 10^3^
	CHP	82 (±14)	0.18 (±0.01)	0.57 (±0.04)	2.0 (±0.2) × 10^3^
	Li-OOH_(1)_	12 (±1)	0.07 (±0.01)	0.21 (±0.01)	6.0 (±0.5) × 10^3^
	Li-OOH_(2)_	7.3 (±0.9)	0.05 (±0.01)	0.19(±0.01)	8.2 (±0.4) × 10^3^
**SeAhpC (Ser)**	H_2_O_2_	178 (±14)	0.42 (±0.01)	1.23 (±0.07)	2.3 (±0.1) × 10^3^
	CHP	76 (±10)	0.19 (±0.01)	0.59 (±0.04)	2.5 (±0.1) × 10^3^
	Li-OOH_(1)_	27 (±3)	0.03 (±0.01)	0.38 (±0.02)	4.7 (±0.5) × 10^3^
	Li-OOH_(2)_	4.5 (±0.6)	0.03 (±0.01)	0.09 (±0.01)	7.0 (±0.6) × 10^3^

^a^ The non-linear regression curves are depicted in Figure S1. Hpx = hydroperoxide.

**Table 4:** Summary of second-order rate constants for HsPrx2 and AhpC oxidation and hyperoxidation.

	Peroxide	k_oxi_ (M^−1^s^−1^)	k_hyp_ (M^−1^s^−1^)	Reference
**HsPrx2**	H_2_O_2_	* (0.2–1.3) × 10^8^	^†^ (1.2) × 10^4^;	* [65], ^†^ [66]
	CHP	(2.43 ± 0.05) × 10^8^	(5.91 ± 0.19) × 10^3^	This work (Figure S2)
	^#^ U-OOH	(2.26 ± 0.13) × 10^6^	^##^ ND	[65]
	Li-OOH_(1)_	(1.01 ± 0.20) × 10^7^	(1.26 ± 0.03) × 10^6^	This work
	Li-OOH_(2)_	(2.54 ± 0.18) × 10^6^	(1.70 ± 0.12) × 10^5^	This work
**AhpC**	H_2_O_2_	^‡^ (1.50 ± 0.07) × 10^8^;	(5.44 ± 0.43) × 10^2^	^‡^ *X. fastidiosa* [47], *P. aeruginosa* (This work)
	^#^ U-OOH	(2.30 ± 0.09) × 10^6^	^##^ ND	*X. fastidiosa* [47]
	Li-OOH_(1)_	^##^ ND	(1.48 ± 0.05) × 10^6^	*P. aeruginosa* (This work)
	Li-OOH_(2)_	^##^ ND	(6.97 ± 0.38) × 10^5^	*P. aeruginosa* (This work)

^#^ U-OOH = Urate hydroperoxide. ^##^ ND = not determined. Temperatures used to data acquisition to human Prx2 and AhpC were this work = 10 °C; * [65] Carvalho et al. 2017 = 22 °C; ^†^ [66] Peskin et al. 2013 = 20 °C and ^‡^ [47] Rocha et al. 2021 = 25 °C.

**Table 5:** Molecular interactions among 2-Cys Prxs and lipid hydroperoxides.

Enzyme	Peroxide	DSγ (Å) Best Conformation	ΔG (kcal/mol)	Residues/Interactions
**HsPrx2**	Li-OOH_(1)_	3.0	−5.2	**Apolar** = Pro44’, Leu45’, Phe49’, Val50’, Glu122’, Ile124’, Leu146’, Pro147’, Pro185”, Phe81 */**Polar** = C_P_51’, Arg127’ and Thr48’
	Li-OOH_(2)_	3.4	−5.7	**Apolar** = Pro44’, C_P_51’, Val50’, Leu146’, Pro147’, Val171”, Pro185”/**Polar** = Arg127’
**PaAhpC**	Li-OOH_(1)_	3.5	−5.7	**Apolar** = Cys46’, Glu115’, Leu117’, Asn139’, Val165”, Pro179”, Val 184”, Phe77 */**Polar** = Thr44’, Phe45’, Arg120’, Ser180”
	Li-OOH_(2)_	4.3	−5.0	**Apolar** = Pro40’, Phe45’, Glu115’, Leu117’, Val165”, Pro179”, Val184”, His76*, Phe77 */**Polar** = Thr44’, Asn139’
**SeAhpC**	Li-OOH_(1)_	3.6	−6.6	**Apolar** = Pro42’, Phe47’, Val48’, Asp114’, Ala140’, Val167”, Pro181”, Gly182”, Phe79 */**Polar** = Ser46’, Cys49’, Arg122’, Asp141’
	Li-OOH_(2)_	3.5	−6.4	**Apolar** = Phe40’, Pro42’, Val48’, Leu119’, Arg122’, Ala140’, Asp141’, Phe79 */**Polar** = Ala43’, Ser46’, Phe47’, Cys49’, Asp114’, Asn139’

The residues of the intimate dimer protomer containing the catalytic triad are marked with prime (’), the C_R_ protomer with quotation (”). Amino acids from the adjacent homodimer are assigned with asterisk (*). DS^γ^ = distance between the proximal oxygen atom of the lipid hydroperoxide and the gamma sulfur atom of C_P_.

## Data Availability

The original contributions presented in this study are included in the article/Supplementary Material. Further inquiries can be directed to the corresponding authors.

## Supplementary Materials

The following supporting information can be downloaded at https://www.mdpi.com/article/10.3390/antiox14121422/s1, Figure S1: Steady-state kinetics of the thioredoxin-linked peroxidase activity of HsPrx2, PaAhpC, and SeAhpC with low concentrations of H_2_O_2_, CHP, Li-OOH(_1_) and Li-OOH(_2_); Figure S2: Determination of second-order rates of HsPrx2 oxidation and hyperoxidation by CHP; Figure S3: Molecular interactions of ligands, HsPrx2 and bacterial AhpC enzymes.
