# Supplementary material for: Linoleic Hydroperoxides Are Potent Hyperoxidative Agents of Sensitive and Robust Typical 2-Cys Peroxiredoxins
Source: Antioxidants (Basel). 2025 Nov 27;14(12):1422. doi: 10.3390/antiox14121422 (PMC12729873; doi:10.3390/antiox14121422)
Supplement: Supplementary file 1 [file antioxidants-14-01422-s001.zip › antioxidants-3947499-supplementary.pdf]

Linoleic hydroperoxides are potent hyperoxidative agents of sensitive and robust typical 2-Cys peroxiredoxins

Supplementary Material

Vitória Isabela Montanhero Cabrera[1†], Sabrina Vargas[1†], Nathália Miranda de Medeiros [2], Gabrielle Nascimento Sividanes[1], Laura Fernandes da Silva[1], Larissa Regina Diniz[2], Thiago Geronimo Pires Alegria[3], João Henrique Ghilardi Lago[4], Marcos Hikari Toyama[1], Sayuri Miyamoto[2], Daniela Ramos Truzzi[2], Luis Eduardo Soares Netto [3*] and Marcos Antonio de Oliveira[1,*]

[1]Instituto de Biociências, Universidade Estadual Paulista, UNESP, São Vicente, SP, Brazil.
[2]Departamento de Bioquímica, Instituto de Química, Universidade de São Paulo, São Paulo, SP, Brazil.
[3]Departamento de Genética e Biologia Evolutiva, Instituto de Biociências, Universidade de São Paulo, São Paulo, SP, Brazil.
[4]Centro de Ciências Naturais e Humanas, Universidade Federal do ABC, Santo André, SP, Brazil

[†] These authors contributed equally to this work.

* Correspondence: marcos.a.oliveira@unesp.br, Tel.: +55 (13) 35697148; nettoles@ib.usp.br, Tel.: +55 (11) 30917589

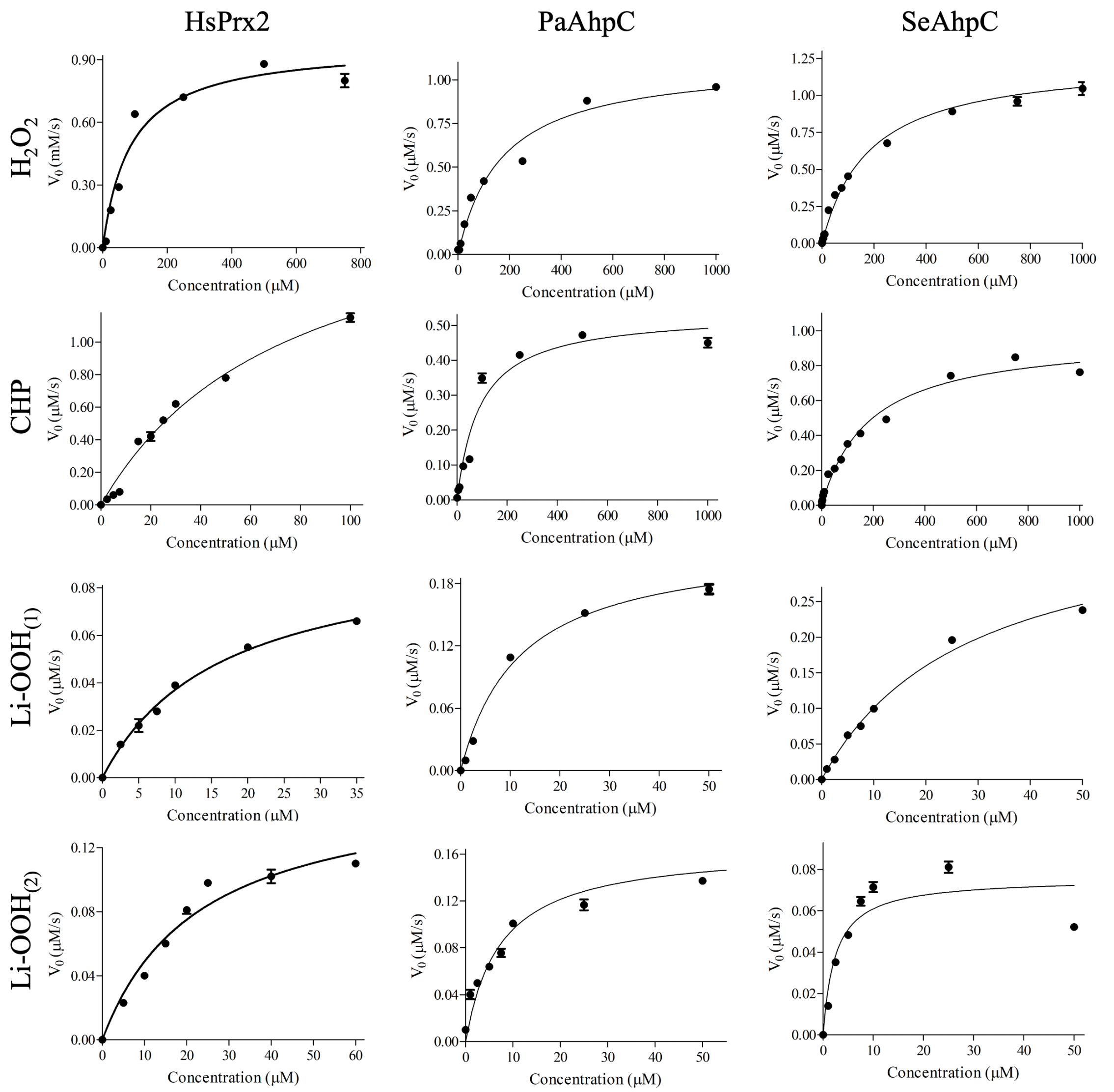

Figure S1. Steady-state kinetics of the thioredoxin-linked peroxidase activity of HsPrx2, PaAhpC, and SeAhpC with low concentrations of H_2O_2, CHP, Li-OOH$_{(1)}$ and Li-OOH$_{(2)}$. Representative peroxidase activity assays were monitored spectrophotometrically by NADPH consumption at 340 nm. Initial rates were plotted against peroxide concentration, and the data were fitted by non-linear regression to the Michaelis-Menten equation to obtain the kinetic parameters (see Table 3, main article). Standard reaction conditions for AhpC enzymes were: 3.0 μM AhpC, 6.0 μM EcTrx, 0.9 μM EcTrxR, 150 μM NADPH, 50 mM HEPES (pH 7.4), 100 μM DTPA, and 1 mM sodium azide. Reactions for HsPrx2 were performed with the yeast thioredoxin system: 3.0 μM HsPrx2, 6.0 μM ScTrx1, 0.9 μM ScTrxR1, 150 μM NADPH, 50 mM HEPES (pH 7.4), 100 μM DTPA, and 1 mM sodium azide. All experiments were performed in triplicate with consistent results.

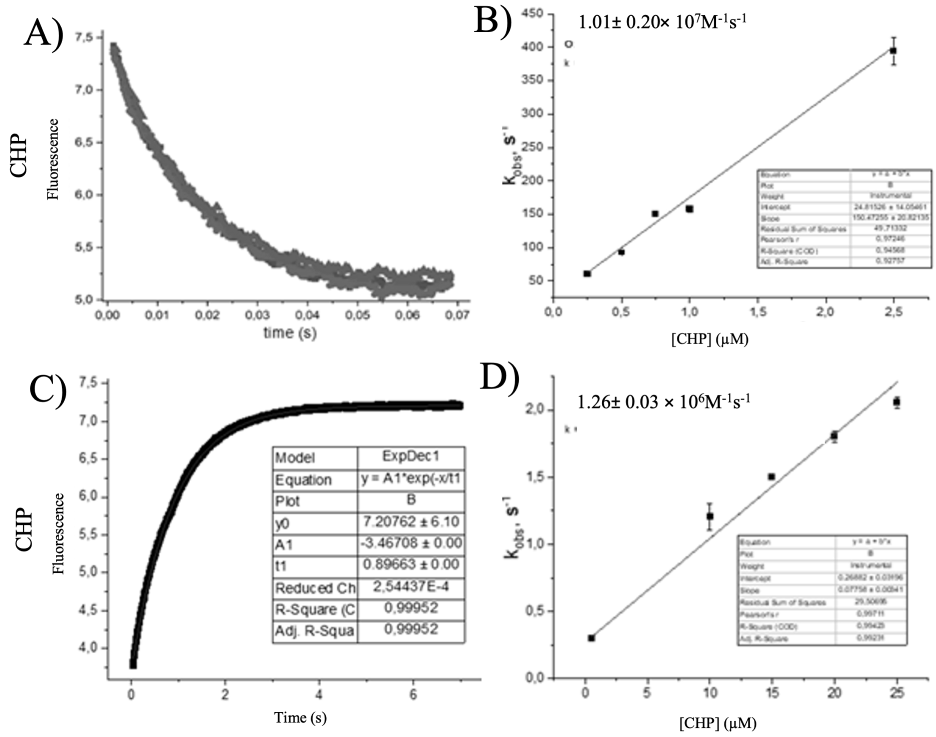

Figure S2. Determination of second order rates of HsPrx2 oxidation and hyperoxidation by CHP. The HsPrx2 enzyme was previously reduced using 5 mM DTT at 37°C for approximately 1 hour. The DTT was removed using a PD-10 desalting column (Cytiva) and argon was pumped into the samples to remove the O_2 and prevent oxidation of the enzyme. The effectiveness of enzyme reduction was then checked using the DTNB assay and 0.5 μM of reduced HsPrx2 (in buffer: 50 mM Tris, pH 7.4 containing 50 mM NaCl) was mixed with CHP in stopped flow equipment (Applied Photophysics SX 17MV spectrofluorometer coupled to stopped flow). The intrinsic fluorescence change reaction of the protein was monitored at (λex = 280 nm; λem = 330 nm). The graphics show the fluorescence profiles of HsPrx2 (fixed concentration of 0.5μM) oxidized with 5μM CHP. In figure (**a)** the oxidation profile of the enzyme by CHP is shown, while in (**c**) is the hyperoxidation profile of HsPrx2. All experiments were repeated 3 times and carried out at 10° C in triplicate. The apparent second order rate constants were determined from the slope of *kobs* values plotted against growing hydroperoxide concentrations. In (**b**) the $k_{CHP\_oxidation}$ and (**d**) $k_{CHP\_hyperoxidation}$ graph. The OriginLab Software was used to perform the calculations of the constants.

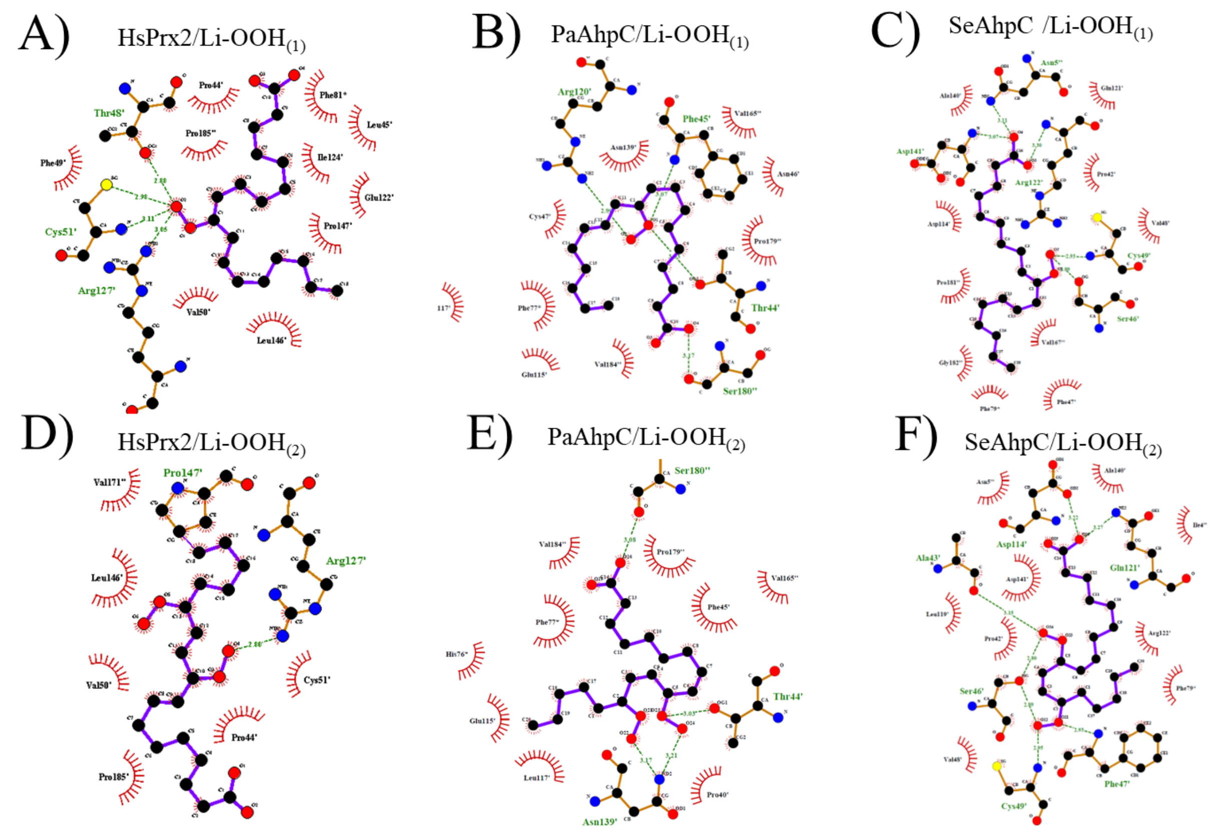

Figure S3. Molecular interactions of Lp-OOHs with HsPrx2 and bacterial AhpC enzymes. Panels (A-C) show the binding of Li-OOH$_{(1)}$ to (A) HsPrx2, (B) PaAhpC and (C) SeAhpC. Panels (D-F) show the binding of Li-OOH$_{(2)}$ to (D) HsPrx2, (E) PaAhpC, and (F) SeAhpC. The 2D interaction diagrams, generated using LigPlot^{+}, depict polar interactions (green dashed lines) and hydrophobic contacts (red arcs with radiating spikes). Residues involved in polar interactions are labeled in green, with bond distances (Å) indicated.
